# Ball Detection Using Deep Learning Implemented on an Educational Robot Based on Raspberry Pi

**DOI:** 10.3390/s23084071

**Published:** 2023-04-18

**Authors:** Dominik Keča, Ivan Kunović, Jakov Matić, Ana Sovic Krzic

**Affiliations:** University of Zagreb, Faculty of Electrical Engineering and Computing, 10000 Zagreb, Croatia; dominik.1acek@gmail.com (D.K.); ivan.kunovic@fer.hr (I.K.); jakov.maticch@gmail.com (J.M.)

**Keywords:** convolutional neural networks, Hough transform, semantic segmentation, object detection, Raspberry Pi, RoboCupJunior, U-NET architecture

## Abstract

RoboCupJunior is a project-oriented competition for primary and secondary school students that promotes robotics, computer science and programing. Through real life scenarios, students are encouraged to engage in robotics in order to help people. One of the popular categories is Rescue Line, in which an autonomous robot has to find and rescue victims. The victim is in the shape of a silver ball that reflects light and is electrically conductive. The robot should find the victim and place it in the evacuation zone. Teams mostly detect victims (balls) using random walk or distant sensors. In this preliminary study, we explored the possibility of using a camera, Hough transform (HT) and deep learning methods for finding and locating balls with the educational mobile robot Fischertechnik with Raspberry Pi (RPi). We trained, tested and validated the performance of different algorithms (convolutional neural networks for object detection and U-NET architecture for sematic segmentation) on a handmade dataset made of images of balls in different light conditions and surroundings. RESNET50 was the most accurate, and MOBILENET_V3_LARGE_320 was the fastest object detection method, while EFFICIENTNET-B0 proved to be the most accurate, and MOBILENET_V2 was the fastest semantic segmentation method on the RPi. HT was by far the fastest method, but produced significantly worse results. These methods were then implemented on a robot and tested in a simplified environment (one silver ball with white surroundings and different light conditions) where HT had the best ratio of speed and accuracy (4.71 s, DICE 0.7989, IoU 0.6651). The results show that microcomputers without GPUs are still too weak for complicated deep learning algorithms in real-time situations, although these algorithms show much higher accuracy in complicated environment situations.

## 1. Introduction

RoboCupJunior (RCJ) [1] is a global-scale, project-oriented educational initiative that sponsors various robotic events for young students. Every year, students are presented with a series of tasks that give them a better insight into mobile robotics and programming. The idea is to program robots to solve different problems: line following, maze escape and robots’ soccer. The final stage in the line-following assignment is a ball-finding task. Once the robot locates the ball inside the bounded area, it must navigate towards it and perform a suitable action (e.g., pick it up). The balls are silver, reflect light and are randomly placed inside the bounded area. As far as we know, no other team has approached this problem using deep learning models.

The ball-finding and tracking problems are very general, and many different methods tackle these tasks. One approach is to use traditional computer vision and image processing algorithms. For example, these algorithms can be utilized to extract colors related to a ball and find circles using the circle Hough transform [2]. While this solution puts the main focus on the ball itself, some techniques use probabilistic algorithms to extract the background and track a ball’s position [3]. The second approach to find balls in images and videos is implementing deep learning algorithms with different neural network architectures, e.g., YOLO and Masked R-CNN, for segmentation and the target objects’ detection [4]. However, in all mentioned papers the ball was a soccer ball or handball, or the implementation was not on a small microcomputer such as Raspberry Pi.

In this preliminary study, we combine computer vision and educational mobile robotics control to solve the problem of finding balls wrapped in aluminum foil in the images and the real-time situations. We analyze and show when the accuracy of deep learning algorithms outclasses the ‘traditional’ computer vision algorithms and when not. Moreover, we demonstrate how complex neural networks can be implemented on Raspberry Pi and combined with robot movement control algorithms. The following are the anticipated contributions of the presented research:

1. A handmade dataset of images of silver balls in different light conditions and surroundings, their annotations for object detection and semantic segmentation;
2. A thorough analysis of Faster R-CNN architecture with different CNN models, U-NET architecture with different encoders and Hough transform performances on test dataset on Google Colab and Raspberry Pi;
3. Educational mobile robot performance analysis in the sense of accuracy and speed when finding silver balls.

The present study is structured as follows. Section 2 provides a short overview of related work. Section 3 gives the model design and architecture of used networks. Section 4 provides data preparation and Section 5 implementation details. Section 6 gives an overview of used evaluation metrics and loss functions. Results are given in Section 7, discussion in Section 8 and the conclusion in Section 9.

## 2. Related Work

Machine learning is an area of research that records constant development and attracts attention. The main characteristic of machine learning algorithms is the improvement of their performances with gained experience. This process is similar to how humans learn and approach solving problems [5]. Deep learning algorithms are a fraction of machine learning algorithms designed for features extraction and recognition in the input data [6]. Deep neural networks are the deep learning algorithms that extract features through a series of layers. The extracted features’ quality depends on the layers’ layout, complexity and architecture. Due to technological development, the complexity of these algorithms is no longer an obstacle for implementation. Therefore, deep learning algorithms are slowly replacing so-called ‘traditional’ algorithms that are not dependent on the learning process. Today, machine learning algorithms are implemented in various devices and applications such as computer vision [7], natural language processing [8] and speech recognition applications [9]. In these areas of research, the algorithms can be adjusted to solve different tasks: object detection [10], semantic segmentation [11], image coloring [12], image reconstruction [13], machine translation [14], sentiment detection [15] and phoneme classification [16]. Autonomous driving [17] and medical imaging [18] are the two popular areas of research that use semantic segmentation. Object detection is applied in various automotive industry applications, e.g., vehicle counting [19].

One approach similar to ours is the one that uses the R-CNN architecture to detect simple-shape objects and later uses this information to control robot arm movements [20]. The second similar project includes Raspberry Pi and a mobile robot. The robot is programmed to follow a ball using a color-based computer vision algorithm implemented on Raspberry Pi [21]. Raspberry Pi is a single-board computer practical for numerous project implementations. Thanks to the various connectors and the simple algorithm execution process, Raspberry Pi can easily be combined with other devices (e.g., mobile robots). Due to constant deep learning algorithms development, their implementation on Raspberry Pi is becoming more frequent. Many performances analyses of deep learning libraries (e.g., PyTorch and TensorFlow) on Raspberry Pi in different applications were published, such as the Internet of Things (IoT) [22] and image classification [23].

Barry et al. developed xYOLO, a neural network-based model for real-time object detection in humanoid soccer [24]. Their work achieved a 0.6675 mAP and 0.68 F1-score at 9.66 FPS on a Raspberry Pi 3 B with 1 GB RAM, demonstrating the potential of deep learning-based object detection in soccer applications on low-end hardware.

Sachdeva proposed a non-neural network-based approach for real-time squash ball detection using size-based segmentation, region-based filtering, and velocity-based constraints with hardware acceleration [25]. The system achieved a notable 0.85 F1-score at 5 FPS on a Raspberry Pi 3B+, proving the viability of non-deep learning methods for detecting fast-moving objects such as squash balls.

Zhangy et al. addressed real-time golf ball detection using convolutional neural networks (CNNs) [26]. They experimented with Faster R-CNN, YOLOv3 and YOLOv3 tiny, achieving the best performance with YOLOv3 tiny at 0.7839 mAP and 357.85 FPS on an Nvidia Titan GPU with 12 GB RAM. Although their work highlights the effectiveness of CNNs in detecting small, fast-moving objects, it differs from our focus on low-end hardware platforms. In summary (Table 1), these related works demonstrate the potential of both neural network-based and non-neural network-based approaches for real-time object detection of balls, with varying levels of accuracy and speed depending on the hardware and algorithms employed.

## 3. Model

**Figure 1:**
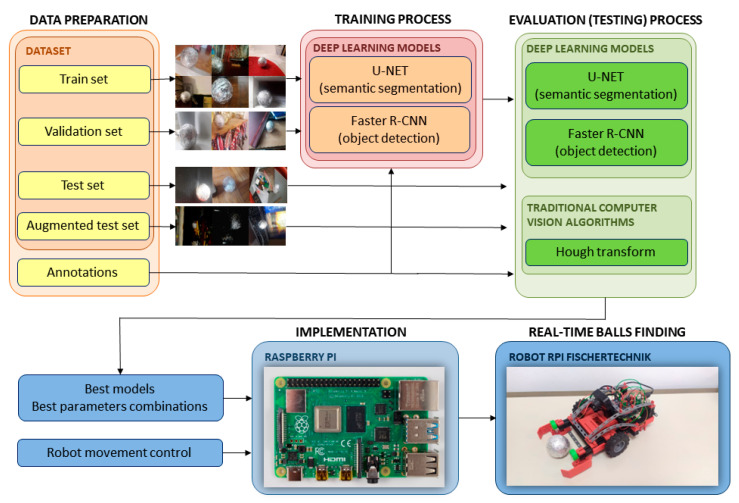
Model for real-time ball finding using educational robot.

The problem of finding balls in the images and videos is tackled using deep neural networks and Hough transform algorithms (Figure 1). We trained deep neural networks to solve two computer vision tasks: semantic segmentation (using U-NET deep convolutional neural network [28]) and object detection (using Faster R-CNN model). The Hough transform is an example of a ‘traditional’ computer vision algorithm. After the training process of the deep learning algorithms, we compared and analyzed the results on the evaluation dataset. Once we extracted the best parameters combination for the Hough transform algorithm and saved deep learning models with trained parameters, we implemented all algorithms on Raspberry Pi. In combination with the robot movement control algorithm, the Fischertechnik robot tried to find the balls in real-time [29]. In real-time robot movement analysis, we compared efficiency and the algorithms’ execution speed. In the following subsections, neural network architectures and problems which they tackled are briefly described.

### 3.1. Semantic Segmentation and U-NET Architecture

Semantic segmentation is a pixel-level classification problem where a neural network assigns one class (in our case a ball) to the pixels in the input image that represent the part of a ball, while the pixels that do not describe a ball are assigned the other class (here background).

**Figure 2:**
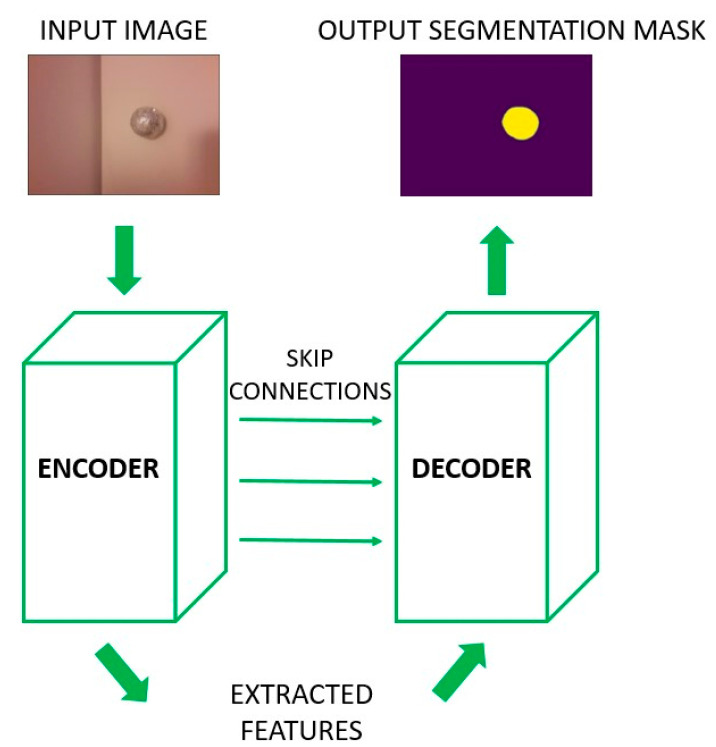
U-NET neural network architecture.

U-NET is a deep convolutional neural network architecture for semantic segmentation (Figure 2). The name U-NET comes from the architecture design that looks like a letter U. The entire network design can be divided into two parts. The encoder is the first part and is in control of the feature extraction process. It consists of a series of convolutional and subsampling layers that minimize the dimensions of the input image. This part of the architecture tries to determine the content of an input image based on the extracted features. The features quality is a result of encoder layers’ layout and design. The encoder output is also an input to the second part of the U-NET architecture, a decoder. It controls linking the extracted features with their spatial relations in the image. Hence, the encoder tries to answer the question “WHAT is the content of an image?”, while the decoder answers the question “WHERE is something in an image?”.

### 3.2. Object Detection and Faster R-CNN Architecture

Object detection is a method of detecting objects in the input images. It solves two minimization problems: localization and classification. The former refers to determining a location of an object in an input image. The latter is trying to assign an appropriate class to a detected object. In our task, the located objects are only the balls wrapped inside the aluminum foil, and their assigned class is the “ball”.

**Figure 3:**
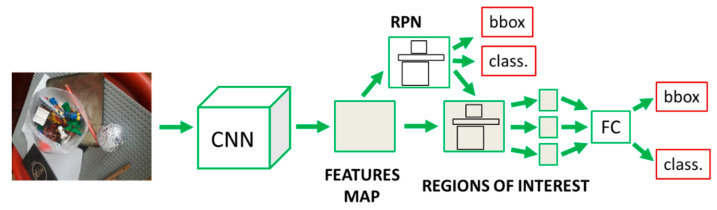
Faster R-CNN architecture.

One way to approach this problem is to separate images in a large number of smaller regions where there is a higher probability for locating the target objects. These areas are called Regions of Interest (ROI). Various image processing algorithms propose their locations. Region-Based Convolutional Neural Networks (R-CNN) solve object detection problems using regions of interest. The main representative models from this group are R-CNN [30], Faster R-CNN [31] and Fast R-CNN [32]. For finding balls in images, we used the Faster R-CNN model. In Figure 3, the features extraction process and model design are demonstrated. An entire input image is prolonged through a CNN to generate a feature map. Region Proposal Network (RPN) is a neural network trained only to create regions of interest from the extracted features. Once the region dimensions are modified, the output fully connected layer uses the extracted feature maps to solve classification and localization problems. In Figure 3, “class.” refers to classification problem, while “bbox” is short for bounding boxes associated with the localization problem. Different CNN backbone models determine the entire networks’ precision and extracted features’ quality.

## 4. Data Preparation

**Figure 4:**
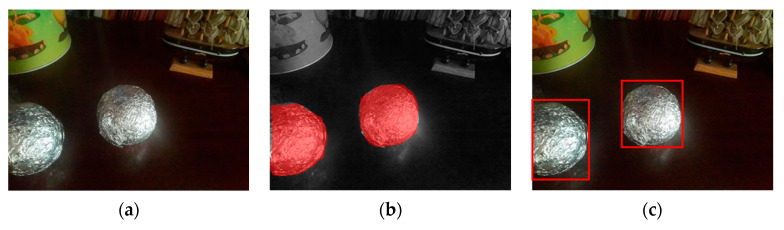
Annotation of images: (**a**) input (original) image, (**b**) annotation for semantic segmentation (red pixels) and (**c**) annotation for object detection (red bounding box).

Input data in all used methods are images, while the outputs are balls’ locations predictions and detected circles. We created a versatile and adequate dataset for training and evaluation. With a mobile phone camera, we took 1200 balls’ images that were all manually annotated. Before the annotation process, we reshaped all the images to the dimensions 600 × 800. For semantic segmentation and Hough transform tasks, annotations are binary masks where the contours of the ball represent the boundaries between the balls and the background (Figure 4a,b). For object detection problems, the coordinates of the bounding boxes around the objects represent the balls annotations (Figure 4c).

The fundamental challenge we were trying to solve was to find a silver ball that was randomly placed inside the bounded area as in the RCJ competition [1]. Therefore, in order to recreate the problem as faithfully as possible, we made the balls of the same size as in the competition and wrapped them in aluminum foil to achieve a reflective silver surface. This reflective surface allowed us to eliminate the color as a feature when using traditional computer vision algorithms and to mainly focus on the shape of a ball. Moreover, since this type of surface to some extent reflects the colors of its surroundings, it brings diversity to data during the training process of deep learning models. 

We took photos of balls in different light conditions and surroundings: in a dark room with little or no light, under indoor lights of different intensities and temperatures (lights temperature range: 3000–5000 K), under daylight, with simple backgrounds and with colorful surroundings full of details. Moreover, we applied random augmentation techniques during the neural network training process. We used augmentation techniques such as rotation, mirroring, luminance changes and optical distortion. Our main goal was to create robust models with good generalization features which would allow neural networks to find balls regardless of surroundings and light conditions.

We also wanted to see how the quality of the training set impacts models’ predictions. Therefore, we created an additional test dataset by applying numerous augmentation techniques on random images, including those techniques that we did not use in the training process, e.g., white noise addition and image blurring. We also annotated every image inside this additional test set. Besides testing and training image sets, we also used a validation set during the training process of deep learning models. The prediction scores on this image set were calculated after every training epoch. We used 70-15-15 (%) as the train-test-validation ratio. The number of images for each set is given in Table 2. The size of the augmented test set is the same as the size of the test set without the augmentation. From then on, we refereed to test dataset without the augmentation as TEST SET and the test set with augmentation as TEST AUG SET.

## 5. Implementation

To run the models and algorithms training and evaluation process, we used the Python programming language within the Google Colab [33] environment and on a Raspberry Pi (RPi) [34]. Image predictions were performed on test images using a Raspberry Pi 400, while the robot’s driving behavior was logged with a Raspberry Pi 4 model B equipped with 2 GB of RAM. For data processing, augmentation, training and testing, we used the following libraries: NumPy (Colab:v1.22.4/RPI:v1.19.5), PyTorch (Colab:v1.10.0+cu111/RPI:v1.11.0a+gitbc2c6ed), PIL (Colab&RPI:v8.4.0), skimage, cv2 (Colab&RPI:v4.6.0), Albumentation (Colab&RPI:v0.4.3), Matplotlib and Catalyst (Colab&RPI:v20.12). To facilitate our research on Raspberry Pi, we employed a pre-installed 64-bit OS image file provided by Qengineering that included versions of PyTorch, Torchvision and several other libraries used for deep learning [35]. This was necessary as there is currently no standard Linux version of PyTorch that is compatible with Raspberry Pi. By utilizing this pre-installed image, we were able to efficiently and effectively carry out our experiments.

The whole process of the deep learning models’ training and their evaluation was conducted using Pytorch and Catalyst libraries. This gave us better insight and more information on data types and sizes during the training and evaluation. Moreover, this enabled us to use output log files to create custom plots and visualization graphs. Since we wanted to stay consistent, we also used PyTorch on Raspberry Pi. To further simplify the learning process of the deep learning model, we used the transfer-learning procedure. The parameters of all pre-trained models are only fine-tuned and adapted to the problem of finding balls.

Segmentation_models.pytorch [36] is a Python deep learning semantic segmentation library that implements numerous pre-trained neural networks, including the U-NET architecture and various pre-trained encoder models. We used the following encoder model families as backbones in the U-NET architecture: ResNet, VGG, EfficientNet, DPN, Inception, MobileNet and DenseNet. Pytorch and Catalyst libraries enable and handle the simple installation of the desired encoders in the U-Net architecture and connection to the decoder side. We used an Adam optimizer [37] with the encoders’ learning rate set to 0.001 and the decoders’ to 0.01. These values were reduced every two epochs during the training process if the metrics stopped improving. During the training all layers were fine-tuned, while the batch-normalization value was left on by default. The entire 20 epochs training process for all the encoder models took 40 h to complete while running on Google Colab GPU. To compare, the training process using CPU took approximately 40 h to train just one VGG encoder.

A Python torch_vision library was used for the object detection. This library implements the Faster R-CNN deep learning models. We compared and analyzed performances of three pre-trained CNN backbone encoders: resnet50_fpn, mobilenet_v3_large_fpn and mobilenet_v3_large_320_fpn. All models are pre-trained on the COCO images dataset [38]. We used a Stochastic Gradient Descent (SGD) optimizer [39] with learning rates set to 0.005 for resnet50_fpn and mobilenet_v3_large_fpn models and to 0.0005 for mobilenet_v3_large_320_fpn. Just like with semantic segmentation models, learning rates were reduced every two epochs when the metrics stopped improving. The training process fine-tuned all of the layers and the batch-normalization was set to frozen by default. It took around 20 h on GPU to train all three pre-trained CNN models.

The Python OpenCV library contains the HoughCircles function that implements the Hough transform algorithm for detecting circles in images. The computational complexity of this function is inferior to that of deep learning models. Therefore, we ran the function evaluation process using the CPU while experimenting with different function input arguments combinations. An entire testing process with all parameter combinations on both TEST and TEST AUG datasets took approximately 10 h to complete.

**Figure 5:**
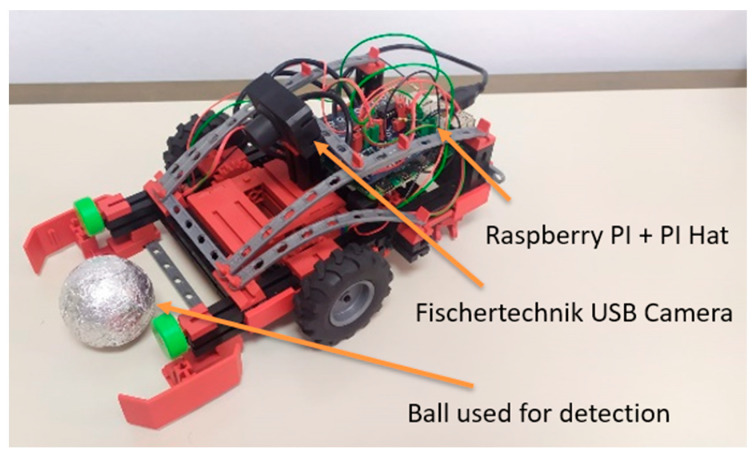
Educational robot with Raspberry Pi 4 Model B used for detection and localization of aluminum balls.

After data preparation, training and evaluation process, chosen algorithms were implemented on an educational robot equipped with two motors, two encoders and a Raspberry Pi Model B [34] for video processing and control of movement (Figure 5). The robot was constructed utilizing the Fischertechnik model TXT Discovery set [40], a Fischertechnik USB camera [41] and a Raspberry Pi single-board computer to facilitate real-time image acquisition and processing. Motors were equipped with Hall-effect sensors as encoders and were connected to a Raspberry Pi using the Pi-F5 interface hardware add-on [42]. External pull-up resistors were added to ensure proper sensor readings. Encoders were utilized to ensure straight-line motion of the robot as motors can occasionally experience mechanical resistance, causing variations in motor speeds and preventing straight-line motion of the robot. The system uses a simple algorithm for straight driving which adjusts the speed of the motors to maintain the desired direction. The algorithm considers the difference in distance traveled by each wheel to adjust the speed of the motors, which allows the robot to maintain a straight path.

The algorithm implements an object detection model, segmentation model and computer vision using Hough’s transform for identifying the position and size of a ball in real-time using a camera feed. The detected ball’s location is used to control the motors of a robotic ball catcher, which moves the device to the ball’s position and attempts to catch it. The algorithm uses a pre-trained model or Hough’s transform, which is fine-tuned for ball detection. The motor control is achieved by computing the position of the ball relative to the center of the camera’s field of view and using this information to steer the robot’s movement left or right and move forward or backward. The algorithm’s performance is evaluated based on the accuracy of the ball detection and the precision of the motor control.

## 6. Evaluation Metrics and Loss Functions

Evaluation metrics are numerical indicators of the quality of the prediction models or algorithms in solving a task. In a ball-finding problem, metrics were calculated based on the ratio of the predicted ball position to the actual ball position of a given annotation process. Accuracy measures used include the DICE coefficient (F1 measure), Intersection over Union (IoU) and precision and recall. Precision and recall are evaluation metrics that provide information on the success of the prediction and the relationship between predicted values (model output) and ground truth values:

(1) $$\mathrm{precision}=\frac{\mathrm{TP}}{\mathrm{TP}+\mathrm{FP}},$$

(2) $$\mathrm{recall}=\frac{\mathrm{TP}}{\mathrm{TP}+\mathrm{FN}},$$

where TP refers to True Positives, FP to False Positives and FN to False Negatives. These metrics were used to calculate the DICE coefficient. The DICE coefficient, also known as the F1 score, is a measure of the similarity of two samples. In the problem of finding balls, the DICE coefficient shows the similarity of the prediction mask and the mask of the actual position of the balls. It can be calculated with precision and recall metrics:

(3) $$\mathrm{DICE}=2\cdot \frac{\mathrm{precision}\cdot \mathrm{recall}}{\mathrm{precision}+\mathrm{recall}}.$$

Like the DICE coefficient, the Jaccard index, or IoU, is an example of an evaluation metric of similarity between samples. It is defined as a division of the size of the intersection and the union of the prediction and ground truth masks:

(4) $$\mathrm{IoU}=\frac{\mathrm{TP}}{\mathrm{TP}+\mathrm{FP}+\mathrm{FN}}.$$

The loss function is a mathematical expression that should be minimized through the training process of the deep learning models. The functions vary depending on the complexity, interpretation and approach to the problem itself. We defined different minimization functions for semantic segmentation and object detection problems. 

To describe the semantic segmentation error function, we combined three losses: DICE loss (DL), Jaccard loss (IoUL) and Binary cross-entropy loss (BCE) with the sigmoid output function σ(x):

(5) DL(x,y)=1−DICE(x,y),

(6) IoUL(x,y)=1−IoU(x,y),

(7) BCE(x,y)=−(y⋅log(σ(x))+(1−y)⋅log(1 – σ(x))),

where *x* represents the model’s prediction and *y* denotes a ground truth value. The final expression for the semantic segmentation minimization function is the combination of all three losses:

(8) L(x,y)= DL(x,y)+IoUL(x,y)+0.8⋅BCE(x,y).

For the object detection problem, we used Faster R-CNN architecture which implements four different loss functions: network classification loss (CLASS), network localization loss (LOCAL), RPN localization loss (RPN) and classification certainty (objectness) loss (OBJECT). The final loss function is:

(9) L=CLASS+RPN+LOCAL+OBJECT.

## 7. Results

The results are compiled from: (1) algorithms’ comparisons of different approaches in the balls’ finding task and (2) performance analysis when algorithms were implemented on Raspberry Pi and tested in real-time while controlling robot movements.

### 7.1. Semantic Segmentation

**Figure 6:**
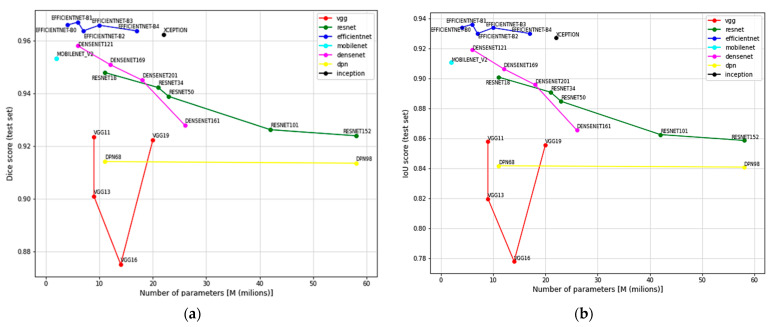

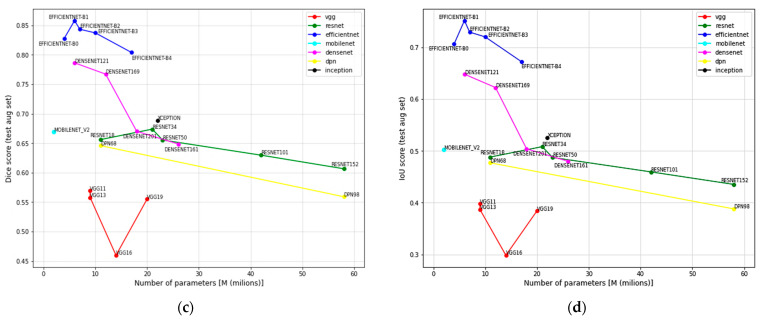
Comparison of different U-NET encoder models’ DICE and IoU evaluation scores on TEST (**a**,**b**) and TEST AUG sets (**c**,**d**).

**Figure 7:**
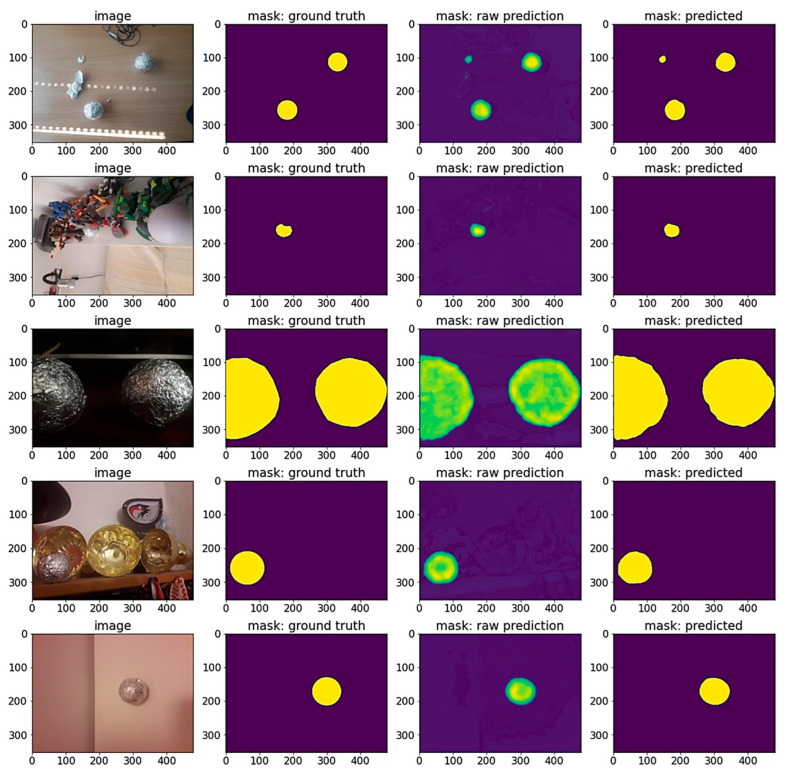
EfficientNet encoder predictions. From left to right: original input image, annotated ground truth mask, raw model’s output prediction and model’s prediction. Mask is represented with yellow color and background with purple color. In the mask: raw prediction column, multiple shades of color correspond to the value of probability at the output of the network: lighter colors correspond to higher probabilities that pixel is part of mask, while darker colors correspond to lower ones.

U-NET architecture with different encoders is used to solve the semantic segmentation problem. In total, 18/19 encoders from seven architecture families were trained: ResNet, VGG, EfficientNet, DPN, Inception, MobileNet and DenseNet. Since Semantic segmentation is trying to solve a pixel-level classification problem on the input images, the output is a 2D prediction array containing the probability that pixels can be assigned to a certain class. We refer to this output as the raw prediction. The prediction mask is then fed to a Sigmoid activation function with a threshold value of 0,5. The pixels with a probability value higher than the threshold are assigned the value of 1, while all the other pixels are set to 0. This binary 2D array is the final processed output of the network referred to as the mask: predicted. In Figure 6, the prediction scores of all the encoders on TEST and TEST AUG datasets using DICE and IoU evaluation metrics on different number of parameters in millions are compared. Every encoder family is marked with a different color. The results show that EfficientNet encoders obtained the highest prediction scores on both test datasets. The exact EfficientNet models’ evaluation values are highlighted in Table 3. In Figure 7, some EfficientNet model predictions are visualized. All models show higher scores on the TEST SET.

### 7.2. Object Detection

**Figure 8:**
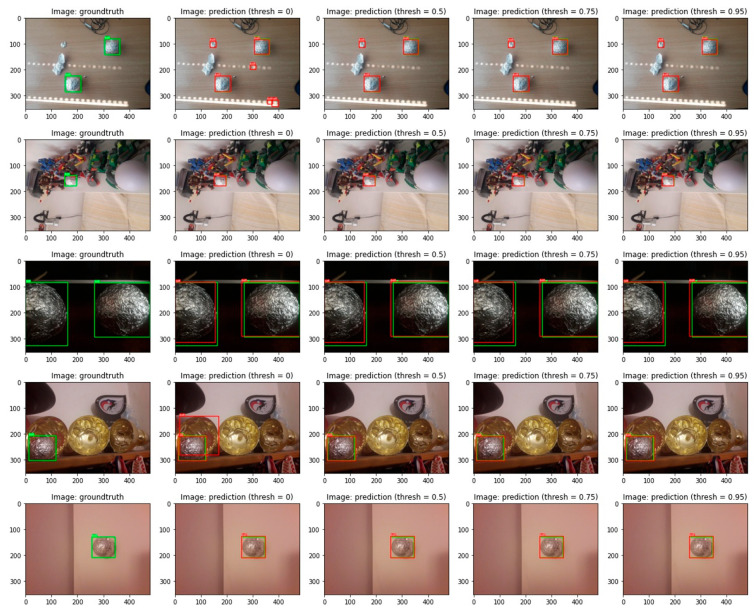
Object detection MobileNet_V3_Large model’s predictions with different probability thresholds. From left to right: original input image, prediction with 0% threshold, prediction with 50% threshold, prediction with 75% threshold and prediction with 95% threshold. Green bounding boxes represent annotated ground truths and the red ones predicted objects.

Faster R-CNN object detection models outputted three parameters during evaluation: bounding box coordinates, class name and probability value. The probability value indicates the model’s certainty that the detected object belongs to the predicted class. In the ball’s detection problem, this value represents models’ certainty that the returned bounding box represents a ball. Lower probability threshold values might result in fake balls detection, while the higher thresholds might omit correct balls predictions. Consequently, evaluation results vary when the different thresholds are applied to the prediction probability value. The evaluation results for three CNN backbone models (resnet50_fpn, mobilenet_v3_large_fpn and mobilenet_v3_large_320_fpn) with different thresholds are demonstrated in Table 4 and Table 5. Table 4 shows DICE and IOU metrics with a 50% probability threshold and Table 5 contains evaluation metrics values with a probability threshold of 95%. Prediction scores in both tables are similar. In other words, predicted bounding boxes probabilities on both test datasets are high enough to satisfy both thresholds. All models obtained higher scores on TEST SET. Figure 8 represents models’ predictions when different thresholds were applied. The first row shows how a lower prediction probability value resulted in fake ball detection.

### 7.3. Hough Transform

**Figure 9:**
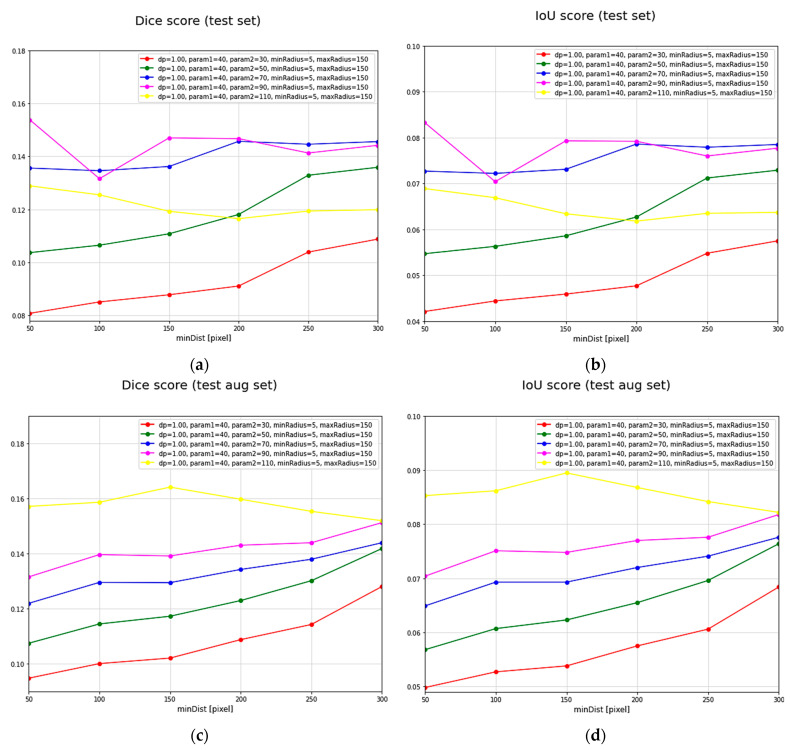
Comparison of DICE and IoU evaluation scores on TEST and TEST AUG sets for different Hough transform parameters’ combinations: (**a**) DICE score for test set, (**b**) IoU score for test set, (**c**) DICE score for augmented test set, and (**d**) IoU score for augmented test set.

To evaluate the Hough transform algorithm, different input arguments were tested. These arguments represent the constraints that dictate the density and the number of detected circles in the images. Different arguments combinations can result in fake or skipped detections. Hough transform DICE and IoU metric scores on TEST and TEST AUG datasets are given in Figure 9. DICE and IoU values are set on the *y*-axis, while the minimum allowed distance between detected circles changes alongside the *x*-axis. Different colors represent different fixed arguments combinations. In Table 6, we highlight arguments’ combinations with the best evaluation results on both test sets.

**Figure 10:**
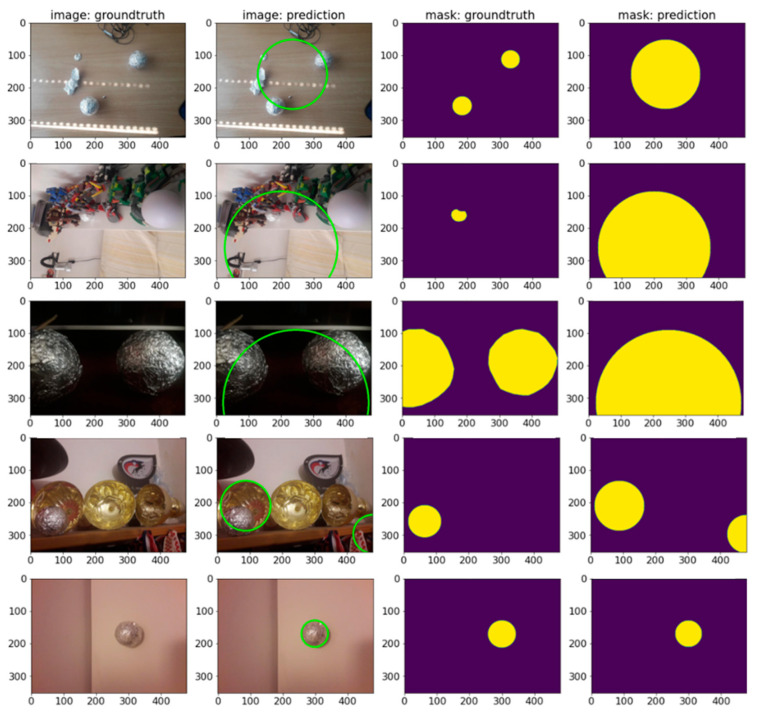
The Hough transform parameters’ (Hough 50-40-50) combination predictions. From left to right: original input image, input image with detected circles, ground truth mask and model’s prediction.

The main reasons for Hough transform’s low evaluation scores are (1) the dynamic background with round objects results in fake circles detection, (2) a wrapped aluminum structure combined with dynamic background results in no detections, (3) one function arguments’ combination might be compatible for some images, but, because of the test sets’ diversity, that combination might not be suitable for the entire dataset, and (4) the implemented Hough transform algorithm works with grayscale images, thus ignoring RGB color components that could be useful for detection. Algorithm predictions where the described problems occurred are visualized in Figure 10.

### 7.4. Algorithm’s Comparison

Although semantic segmentation and object detection are both deep learning problems, their core procedures to find the balls in the images differ. While the first implements the pixel-level classification, the latter performs both classification and localization on the region’s level. Consequently, the ways these methods minimize their loss functions are also different. Unlike the deep learning algorithms that try to find balls in images based on the extracted features, the Hough transform algorithm finds circles in images. Moreover, this traditional approach does not consider RGB color components, only grayscale image representations.

**Figure 11:**
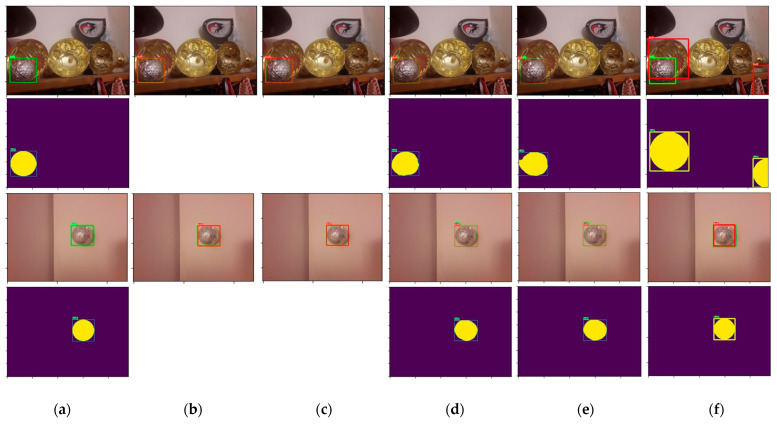
Comparison of predictions with bounding boxes. From left to right: (**a**) ground truth, (**b**) object detection MOBILENET_V3_LARGE_320 with threshold 95%, (**c**) object detection RESNET50 with threshold 95%, (**d**) semantic segmentation EFFICIENTNET_B0, (**e**) semantic segmentation MOBILENET_V2, (**f**) Hough 50-40-50. The first and third rows are images with bounding boxes, where green bounding boxes represent annotated ground truths and the red ones predicted objects. The second and fourth rows are masks with bounding boxes, where yellow pixels represent masks, and green or yellow lines represent bounding boxes. For object detection, masks were not created.

To compare the used methods, we created bounding boxes around semantic segmentation, Hough transform prediction and ground truth masks using Python’s OpenCV library. We utilized the library’s functions that track contours in binary images and draw approximated bounding rectangles (boxes). By creating bounding boxes, we obtained the binary masks that correspond to the masks used in the object detection process. DICE and IoU scores were calculated on those bounded box masks generated from ground truth and prediction images. We used only TEST SET for testing since in robot implementation we will not have such a complicated situation as in TEST AUG SET. Examples of comparison of prediction with bounding boxes for different methods are given in Figure 11.

We consider that model or arguments combinations are optimal if they obtain the highest DICE and IoU scores on test datasets. Further, we measured average execution times for each method. Subsequently, we conducted tests on the Raspberry Pi using the same images and methods. All results are given in Table 7. The table also includes information on the ranking of the algorithms based on the DICE score on Raspberry Pi and Colab, as well as the time for processing images on Raspberry Pi and Colab.

Once we calculated the evaluation metrics scores, we highlighted the best deep learning models and arguments combinations for all used algorithms. It is important to note that creating bounding boxes and recalculating evaluation metrics does not overcome the difference between methods. This procedure allowed us just to compare results. Based on the results, we chose five algorithms that were implemented to track the ball on the robot: the Hough transform (Hough 50-40-50) as the traditional computer vision algorithm, RESNET50 and MOBILENET_V3_LARGE_320 as most accurate and fastest object detection algorithms and EFFICIENTNET-B0 and MOBILENET_V2 as most accurate and fastest segmentation algorithms.

### 7.5. Robot Performance Analysis

**Figure 12:**
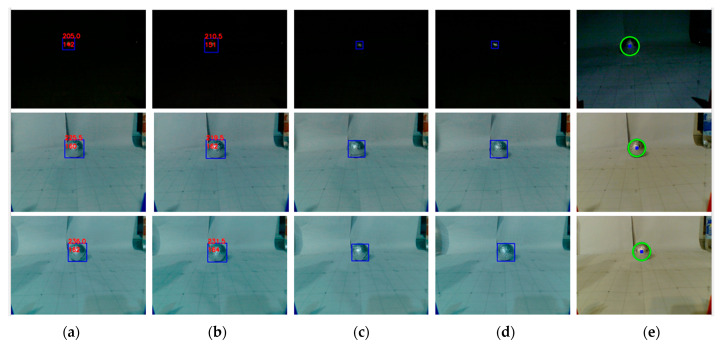
Comparison of predictions of the robot. From left to right: (**a**) MOBILENET_V3_LARGE_320, (**b**) RESNET50, (**c**) EFFICIENTNET-B0, (**d**) MOBILENET_V2, (**e**) Hough 50-40-50 transform. Differences in white balance in the last column are the result of the OpenCV library. Blue bounding boxws represent predicted objects using deep learning methods, and green circle represent predicted objects using Hough transform.

After selecting algorithms, experiments were conducted with the educational robot and with balls placed in six different positions: distances of 30 and 40 cm from the robots’ camera and in three horizontal positions: left, center and right. The left and right positions were shifted 7.5 cm from the center of the central line of the ball. We repeated the experiment with different light conditions: one bulb, two bulbs and a mobile phone flashlight. Images with different light conditions at a distance of 40 cm from the robot and predictions for all observed methods are given in Figure 12.

We logged all robots’ drives and measured the time from program startup to arrival to the ball. The moment of arrival at the ball was the end of each drive (the robot stopped in front of the ball). We measured the time it took to initialize the network, the time it took to predict each image, the time it took to reach the ball and the total time. All average times are given in Table 8. Moreover, Table 8 includes memory usage as well as accuracy measured by the DICE coefficient and IoU.

The Hough 40-90-50 transform had the best results in Table 7 but showed poor results when tested with the educational robot in white surroundings and could not detect any balls. However, the Hough 50-40-50 showed very high DICE and IoU when implemented on the educational robot and therefore it is used in further analysis and given in Table 8 and Figure 12.

Additionally, the Hough transform was the fastest method, with a full time of 4.71 s, followed by MOBILENET_V3_LARGE_320 and EFFICIENTNET-B0. RESNET50 had the slowest full time with 158.71 s. In terms of memory usage, RESNET50 used the most RAM with 458 MB, followed by MOBILENET_V3_LARGE_320, MOBILENET_V2 and EFFICIENTNET-B0, while the Hough transform used only 17.81 MB.

Regarding accuracy, all models achieved high scores, with RESNET50 having the highest DICE coefficient (0.8899) and IoU value (0.8016), followed by MOBILENET_V3_LARGE_320 (DICE = 0.8559, IoU = 0.7482) and the Hough transform (DICE = 0.7989, IoU = 0.6651). EFFICIENTNET-B0 and MOBILENET_V2 achieved slightly lower accuracy scores, with DICE coefficients of 0.7373 and 0.7598, respectively, and IoU values of 0.5839 and 0.6126. The total time for each model’s prediction and the time for initialization, prediction, drive and RAM usage are shown in Table 8.

Overall, the Hough transform method was the fastest and most memory-efficient method, but it achieved slightly lower accuracy than some of the deep learning models.

## 8. Discussion

Obtained results show that deep learning algorithms outperform the Hough transform algorithm on Google Colab and Raspberry Pi on the test set. However, the Hough transform proved elusive when used on an educational robot under precisely defined conditions in terms of used time and RAM, regardless of the average results in DICE and IoU measures.

High prediction scores on test datasets indicate that all neural network models achieved good generalization features. However, when analyzing Figure 6, we can see that models with higher complexity and more parameters do not necessarily result in better predictions. We can conclude that neural networks’ complexity can cause overfitting and lower prediction scores on a given task. When comparing evaluation results on TEST and TEST AUG datasets, we can see the direct impact of the complex augmentation techniques on models’ performance.

Although neural networks become more accurate with gained experience as the training process progresses, their predictions still depend on the features extraction process, numerical relations and pixel values in the input data. In other words, deep learning algorithms cannot perfectly simulate the human visual system and brain processing. The input image modifications not used in the training process have a direct impact on models’ performances and can result in wrong predictions. On the other hand, these changes do not have a large impact on human perception and image understanding of the image content. Despite that, high prediction scores on test datasets indicate that finding balls in images while using the deep neural networks was completed successfully: all models found the balls wrapped into aluminum foil and differentiated them from the surrounding background. Problems of using deep learning algorithms include the long training process, the redundant data preparation process and the computational complexity of the CNN architectures.

The Hough transform algorithm predictions show low scores on test datasets. The first reason for its poor performances is that it did not return balls’ positions in images, but rather detected circles that satisfied the HoughCircles function argument constraints. The second reason is that this approach works with grayscale input images and not with colored data. In other words, every colored image in the dataset is transformed into its luminance representation, thus losing valuable information. The advantage of this approach is low computational complexity, which allows the algorithm to run in real-time. Moreover, we can evaluate the Hough transform algorithm without the training process.

The results indicate that deep learning algorithms, such as RESNET50 for object detection and EFFICIENTNET-B0 for segmentation, outperform traditional methods, such as the Hough transform, in finding and locating balls. The performance metrics for deep learning algorithms are generally high, with DICE and IoU values above 0.9.

However, the results also indicate that implementing deep neural networks on programmable devices, such as Raspberry Pi and educational robots, can be challenging due to their complexity and limitations in processing power. The processing time on Raspberry Pi is generally much higher than on Colab, and some algorithms, such as EFFICIENTNET-B2, show a significant drop in performance on Raspberry Pi compared to Colab.

Although it was expected that the Hough transform would not be nearly as good as neural networks when implemented on the educational robot, it proved to be sufficiently accurate and useful for the specific, simple task of finding balls against a predominantly white background. As expected, it performed poorly in low light conditions but was unsurpassedly successful in better conditions.

Overall, Table 7 provides valuable insights into the performance of different computer vision algorithms for finding and locating balls and highlights the challenges of implementing deep learning algorithms on programmable devices.

Implementation of these unoptimized networks was too slow for real-time execution on the robot. However, our outreach goal was to test various deep learning methods and to see which of them showed satisfactory results so that we could use them in teaching educational robotics in the future. We focused on pre-trained models available in used libraries. In future research, optimized networks for microcomputers will be tested. Additionally, pruning, sparsing and using integers instead of float memory models could speed up the process on the implementation side. These are next steps that could be tested and compared with precision with current networks.

Besides trying other neural network approaches and architectures, it would also be interesting to experiment with different settings of used networks. During the training process of all models, we mostly utilized the default values of parameters of pre-trained models and libraries’ functions, while the semantic segmentation minimization function was chosen based on experiences in previous projects. In the future, we would like to try different minimization functions and see how their selection affects the optimization of the models’ parameters. In addition, we would like to change some default libraries’ settings during training, such as batch normalization usage and dropout of individual layers.

## 9. Conclusions

In this paper we provided a detailed comparison of deep learning methods (object detection and semantic segmentation) and the traditional Hough transform method on the example of a ball-finding task. The goal was to implement algorithms on an educational robot Fischertechnik controlled with the microcomputer Raspberry Pi so that the robot could find balls wrapped in aluminum foil in a space with a predominantly white background. This kind of task can be found in RobocupJunior competitions and is generally solved using sensors (e.g., ultrasonic sensors). Similar analysis and implementation can be made for situations from real life, for example a robot that finds balls in sports (e.g., golf, tennis, football), applications in building houses, detection of garbage, types of fruit, etc. Some of these applications will be analyzed as possible future research directions.

Although deep learning methods showed multiple times better results on learning, training and validation sets, when implemented on a robot, it turned out that the Hough transform is a good enough method in terms of speed and accuracy. These results were achieved primarily because the robot surroundings were much simpler than the surroundings in images in the learning set. As a conclusion, if we want a high accuracy of searching for the ball in situations with a colorful background or complicated lighting conditions, it is recommended to use object detection (e.g., RESNET50) or semantic segmentation (e.g., EFFICIENTNET-B0) methods. On the other hand, if we have limited resources (e.g., usage of a microcomputer without GPU), the Hough transform with a proper choice of parameters is still a reasonable choice that gives satisfactorily accurate and fast results.

## Figures and Tables

**Table 1:** Summary of related work on ball detection.

Author	Year	Addressed Problem	Dataset	Neural Networks	Proposed Approach	Platform Used	Accuracy and Speed
Speck, D. et al. [27]	2018	Real-world RoboCup Soccer ball detection	Hamburg Bit-Bots Ball Dataset 2018 (>22,000 images)	Yes	Custom fully convolutional neural network (FCNN)	NVIDIA Jetson TX2	FCNN (“Model 2”) 0.743 IoU @ 35 FPS
Barry et al. [24]	2019	Real-time object detection in humanoid soccer on low-end hardware	Custom (1423 images)	Yes	xYOLO	RPi 3 B, 1 GB RAM	0.6675 mAP; 0.68 F1 @ 9.66 FPS
Sachdeva, S. [25]	2019	Real-time squash ball detection on low-end hardware	Images from squash.tv (2500 images)	No	size-based segmentation, region-based filtering and velocity based	RPi 3 B, 1 GB RAM	0,85 F1 @ 5 FPS
Zhang, X. et al. [26]	2021	Real-time golf ball detection	Custom (1699 golf ball images)	Yes	Faster R-CNN YOLOv3 YOLOv3 tiny	Nvidia Titan GPU 12 GB RAM	Faster R-CNN 0.8328 mAP @ 27.78 FPS; YOLOv3 tiny mAP 0.7839 @ 357.85 FPS

**Table 2:** Image sets size.

	Training Set	Validation Set	Test Set without Augmentation	Test Set with Augmentation
Number of images	840	180	180	180
Origin	captured with camera	captured with camera	captured with camera	randomly augmented images

**Table 3:** EfficientNet encoders DICE and IoU scores on TEST and TEST AUG datasets. Best results are bold.

Encoder	DICE (TEST)	IoU (TEST)	DICE (TEST AUG)	IoU (TEST AUG)
EFFICIENTNET-B0	0.9659	0.9341	0.828	0.7065
**EFFICIENTNET-B1**	**0.967**	**0.9361**	**0.8583**	**0.7518**
EFFICIENTNET-B2	0.9637	0.9299	0.8435	0.7294
EFFICIENTNET-B3	0.9658	0.9338	0.8374	0.7203
EFFICIENTNET-B4	0.9637	0.93	0.8041	0.6724

**Table 4:** Faster R-CNN backbone models’ DICE and IoU scores on TEST and TEST AUG set. Probability threshold 0.5 (50%). Best results are bold.

CNN Backbone Model	DICE (TEST)	IoU (TEST)	DICE (TEST AUG)	IoU (TEST AUG)
RESNET50	0.9502	0.905	0.7422	0.59
MOBILENET_V3_LARGE	**0.9621**	**0.9269**	0.7683	0.6238
MOBILENET_V3_LARGE_320	0.9585	0.9203	**0.8041**	**0.6724**

**Table 5:** Faster R-CNN backbone models’ DICE and IoU scores on TEST and TEST AUG set. Probability threshold 0.95 (95%). Best results are bold.

CNN Backbone Model	DICE (TEST)	IoU (TEST)	DICE (TEST AUG)	IoU (TEST AUG)
RESNET50	**0.9755**	**0.9522**	0.6903	0.5271
MOBILENET_V3_LARGE	0.9611	0.9251	0.6685	0.502
MOBILENET_V3_LARGE_320	0.9579	0.9192	**0.7332**	**0.5788**

**Table 6:** Hough transform DICE and IoU scores on TEST and TEST AUG sets for different parameters combinations, where p stands for parameter, minR for min radius and maxR for max radius. Best results are bold.

Hough	dp	p_1_, p_2_	minR, maxR	minDist	DICE (TEST)	IoU (TEST)	DICE (TEST AUG)	IoU (TEST AUG)
Hough 40-110-150	1.00	40, 110	5, 150	150	0.1193	0.0634	**0.1642**	**0.0895**
Hough 40-90-50	1.00	40, 90	5, 150	50	**0.1538**	**0.0833**	0.1316	0.0704
Hough 40-90-150	1.00	40, 90	5, 150	150	0.147	0.0793	0.1392	0.0748
Hough 50-40-50	1.2	50, 40	25,300	50	0.134	0.0718	0.1462	0.0788

**Table 7:** Test results tested on Google Colab and Rasperry Pi (RPi) using bounding boxes around semantic segmentation, Hough transform predictions and ground truth masks. The Hough transform and the two best algorithms based on DICE, IoU and time for object detection and segmentation are in bold and were chosen for implementation in the educational robot.

Type	Net Name	DICE Colab	DICE Rpi	IoU Colab	IoU Rpi	Rank Colab	Rank Rpi	Time Colab, [s]	Time Rpi, [s]	Time Rank Rpi
Obj. det	**RESNET50**	**0.9763**	**0.9763**	**0.9536**	**0.9328**	**1**	**1**	0.16	69.79	26
Segm.	**EFFICIENTNET-B0**	0.9655	**0.9656**	0.9333	**0.9335**	3	**2**	0.10	8.23	5
Segm.	EFFICIENTNET-B1	0.9677	0.9653	0.9373	0.9328	2	3	0.09	9.12	7
Obj. det	MOBILENET_V3_LARGE	0.9616	0.9616	0.926	0.926	5	4	0.09	7.40	3
Segm.	DENSENET121	0.9605	0.9604	0.924	0.9238	8	5	0.10	14.13	14
Segm.	XCEPTION	0.9604	0.9601	0.9238	0.9233	9	6	0.59	17.29	17
Obj. det	**MOBILENET_V3_LARGE_320**	0.9579	0.9579	0.9192	0.9192	10	7	**0.06**	**2.01**	**2**
Segm.	**MOBILENET_V2**	0.9526	0.9521	0.9094	0.9087	11	8	**0.07**	8.05	4
Segm.	DENSENET169	0.9501	0.9488	0.905	0.9026	12	9	0.12	14.60	15
Segm.	DENSENET201	0.946	0.9462	0.8975	0.8978	14	10	0.12	16.76	16
Segm.	RESNET18	0.9462	0.9458	0.8978	0.8971	13	11	0.07	8.93	6
Segm.	RESNET50	0.9404	0.9387	0.8875	0.8846	15	12	0.08	13.58	12
Segm.	RESNET34	0.9388	0.9384	0.8847	0.884	16	13	0.07	10.98	10
Segm.	VGG11	0.9283	0.9269	0.8663	0.8638	17	14	0.07	17.62	19
Segm.	DENSENET161	0.9255	0.9264	0.8613	0.8629	18	15	0.13	23.78	22
Segm.	RESNET101	0.9253	0.9251	0.861	0.8607	19	16	0.09	17.37	18
Segm.	VGG19	0.9241	0.9249	0.8589	0.8602	20	17	0.07	34.29	25
Segm.	RESNET152	0.9241	0.9243	0.8589	0.8593	21	18	0.19	22.11	20
Segm.	EFFICIENTNET-B4	0.9616	0.9193	0.926	0.8507	6	19	0.11	14.04	13
Segm.	EFFICIENTNET-B3	0.9649	0.9191	0.9322	0.8504	4	20	0.09	11.90	11
Segm.	DPN68	0.9139	0.9167	0.8415	0.8463	22	21	0.09	10.94	9
Segm.	VGG13	0.9119	0.9117	0.838	0.8378	23	22	0.07	23.51	21
Segm.	DPN98	0.9108	0.9104	0.8363	0.8355	24	23	0.12	24.77	23
Segm.	VGG16	0.8754	0.8741	0.7784	0.7764	25	24	0.07	29.19	24
Segm.	EFFICIENTNET-B2	0.9613	0.8315	0.9256	0.7116	7	25	0.11	10.13	8
Hough	**Hough 40-90-50**	0.1622	0.1622	0.0883	0.0883	26	26	0.20	**0.24**	**1**
Hough	**Hough 50-40-50**	0.1583	0.1583	0.0859	0.0859	27	27	0.20	**0.43**	**1**

**Table 8:** Results of algorithms running on the educational robot Fischertechnik with Raspberry Pi. Time init represents the average network initialization time, and Time pred is the average time of image prediction. Total time is the sum of times for initialization, prediction, getting images from camera, transformation, targeting and driving forward and saving images. All times are in seconds. DICE and IoU were calculated as a result of three different room light conditions: one bulb, three bulbs and an LED smartphone flashlight including the six positions of the ball.

Encoder	DICE	IoU	Total Time	Time Init	Time Pred	Time Drive	RAM [MB]
MOBILENET_V3_LARGE_320	0.8559	0.7482	12.74	5.33	4.69	2.17	236
RESNET50	0.8899	0.8016	158.71	17.62	148.27	2.18	458
EFFICIENTNET-B0	0.7373	0.5839	26.82	3.00	16.35	2.95	52
MOBILENET_V2	0.7598	0.6126	26.22	3.28	15.28	3.81	55
Hough 50-40-50	0.7989	0.6651	4.71	0.22	0.17	2.44	17.81

## Data Availability

Source code will be made available on request.
